# Integration of a Kalman filter in the geographically weighted regression for modeling the transmission of hand, foot and mouth disease

**DOI:** 10.1186/s12889-020-08607-7

**Published:** 2020-04-10

**Authors:** Bisong Hu, Wenqing Qiu, Chengdong Xu, Jinfeng Wang

**Affiliations:** 1 School of Geography and Environment, Jiangxi Normal University, Nanchang, 330022 China; 2 State Key Laboratory of Resources and Environmental Information System, Institute of Geographic Sciences and Natural Resources Research, Chinese Academy of Sciences, Beijing, 100101 China

**Keywords:** Hand, Foot and mouth disease, Kalman filter, Geographically weighted regression, Spatiotemporal pattern, Determinant factors

## Abstract

**Background:**

Hand, foot and mouth disease (HFMD) is a common infectious disease whose mechanism of transmission continues to remain a puzzle for researchers. The measurement and prediction of the HFMD incidence can be combined to improve the estimation accuracy, and provide a novel perspective to explore the spatiotemporal patterns and determinant factors of an HFMD epidemic.

**Methods:**

In this study, we collected weekly HFMD incidence reports for a total of 138 districts in Shandong province, China, from May 2008 to March 2009. A Kalman filter was integrated with geographically weighted regression (GWR) to estimate the HFMD incidence. Spatiotemporal variation characteristics were explored and potential risk regions were identified, along with quantitatively evaluating the influence of meteorological and socioeconomic factors on the HFMD incidence.

**Results:**

The results showed that the average error covariance of the estimated HFMD incidence by district was reduced from 0.3841 to 0.1846 compared to the measured incidence, indicating an overall improvement of over 50% in error reduction. Furthermore, three specific categories of potential risk regions of HFMD epidemics in Shandong were identified by the filter processing, with manifest filtering oscillations in the initial, local and long-term periods, respectively. Amongst meteorological and socioeconomic factors, the temperature and number of hospital beds per capita, respectively, were recognized as the dominant determinants that influence HFMD incidence variation.

**Conclusions:**

The estimation accuracy of the HFMD incidence can be significantly improved by integrating a Kalman filter with GWR and the integration is effective for exploring spatiotemporal patterns and determinants of an HFMD epidemic. Our findings could help establish more accurate HFMD prevention and control strategies in Shandong. The present study demonstrates a novel approach to exploring spatiotemporal patterns and determinant factors of HFMD epidemics, and it can be easily extended to other regions and other infectious diseases similar to HFMD.

## Background

Hand, foot and mouth disease (HFMD) is a common infectious disease caused by at least 20 enteroviruses including enterovirus 71 (EV-A71) and Coxsackie virus A16 (CA-V16) [1]. HFMD usually affects infants and children under five and its main symptoms include fever, mouth ulcers and blisters or vesicles on the hands, feet, and mouth. Existing vaccines are only partially effective for specific HFMD pathogens [2]. The transmission mechanism of HFMD epidemics is complicated and its spatiotemporal pattern is not yet fully understood [3]. During the last decades, HFMD has been widespread in Asian countries, such as Japan, Malaysia, and Singapore [4–6]. In China, the first large-scale outbreaks of HFMD occurred in Linyi city, Shandong province in 2007 [7] and in Fuyang city, Anhui province in 2008 [8]. Next, in May 2008 the Ministry of Health of China listed HFMD as a statutorily notifiable infectious category C disease. China’s infectious disease automated alert and response system (CIDARS) was developed in the same year for the early detection and rapid response to the outbreaks of infectious diseases, and the system performance was satisfactory in the detection of HFM disease outbreaks, with a sensitivity of 92.7% and a specificity of 95.0% [9]. An extensive three-level HFMD surveillance laboratory network was established in mainland China since 2008, and the surveillance data from 2008 to 2017 indicated a high incidence of HFMD occurred every 2 years and the high-risk regions were located in southern, eastern, and central China [10]. Numerous studies on HFMD epidemics were implemented in various regions, particularly in provinces with serious epidemics, such as Guangdong [11, 12], Sichuan [13, 14], Henan [15, 16], Shandong [17, 18], and others.

Previous studies have mainly focused on characteristics of the epidemic [1, 15, 19], such as spatiotemporal patterns and correlations with various risk factors. HFMD epidemics have significant temporal variations and seasonality features, which vary between regions [20–23]. HFMD epidemics were spatially dispersed across counties in mainland China in the summer and winter, while clustered in spring and autumn; they were also geographically clustered in and closely linked to regions with high levels of monthly precipitation [3, 24]. In addition, HFMD epidemics follow complicated spatiotemporal patterns and transmission mechanisms, and are associated with several types of risk factors. For example, the HFMD incidence in Singapore has been found to be affected in a non-linear manner by the maximum temperature and rainfall, with a time lag of 1–2 weeks, and thresholds of 32 °C and 75 mm, respectively [25]. Furthermore, in Japan and Vietnam, temperature and humidity had significant effects on the HFMD incidence [21, 26]. The spatial variation of HFMD in counties across mainland China was found to be affected by a combination of climate variables, while the spatiotemporal transmission was largely driven by variations in temperature, with a 7-week lag [3]. Extreme precipitation was significantly associated with childhood HFMD in Hefei, China, and the susceptible risk in urban areas was much higher than that in rural ones [27]. High-risk areas of HFMD incidence temporally varied from northeast to southwest in Sichuan, China, and temperature and per capita gross domestic product (GDP) were the main positive driving factors [13].

Non-linear associations have been found between the HFMD incidence and meteorological, land-use, normalized difference vegetation index (NDVI) and socioeconomic factors in Shandong, China [18]. Many other studies have also focused on exploring of HFMD spatiotemporal patterns and the associated driving factors, by considering a variety of methods [3, 11–14, 16, 18, 20, 22–24, 26–34]. However, the measurement and prediction of the HFMD incidence are usually considered separately, and rarely in an integrated fashion. The former is mainly accomplished by using case reports, while the latter requires specific quantitative models. The modeling of HFMD transmission and the corresponding analysis results could be influenced by explanatory variable selection, spatial autocorrelation, spatial stratified heterogeneity, spatiotemporal nonstationary, etc. Excluding the above factors, the unsatisfactory performance of some specific models is probably caused by both the prediction uncertainty and the measurement noise. On the other hand, the measurement and prediction could be combined recursively in the modeling of HFMD transmission. Considering both the measurement noise and the prediction uncertainty can positively improve the estimation accuracy of the HFMD incidence, and could possibly offer a fresh perspective in exploring spatiotemporal patterns and determinant factors of the epidemic. This study aims to estimate the spatiotemporal evolution of the HFMD incidence by districts using a Kalman filter integrated with geographically weighted regression (GWR), to explore the spatiotemporal variation characteristics and potential risk regions, and to quantitatively evaluate the influence of meteorological and socioeconomic factors on the HFMD variation.

## Methods

### Study region

**Fig. 1:**
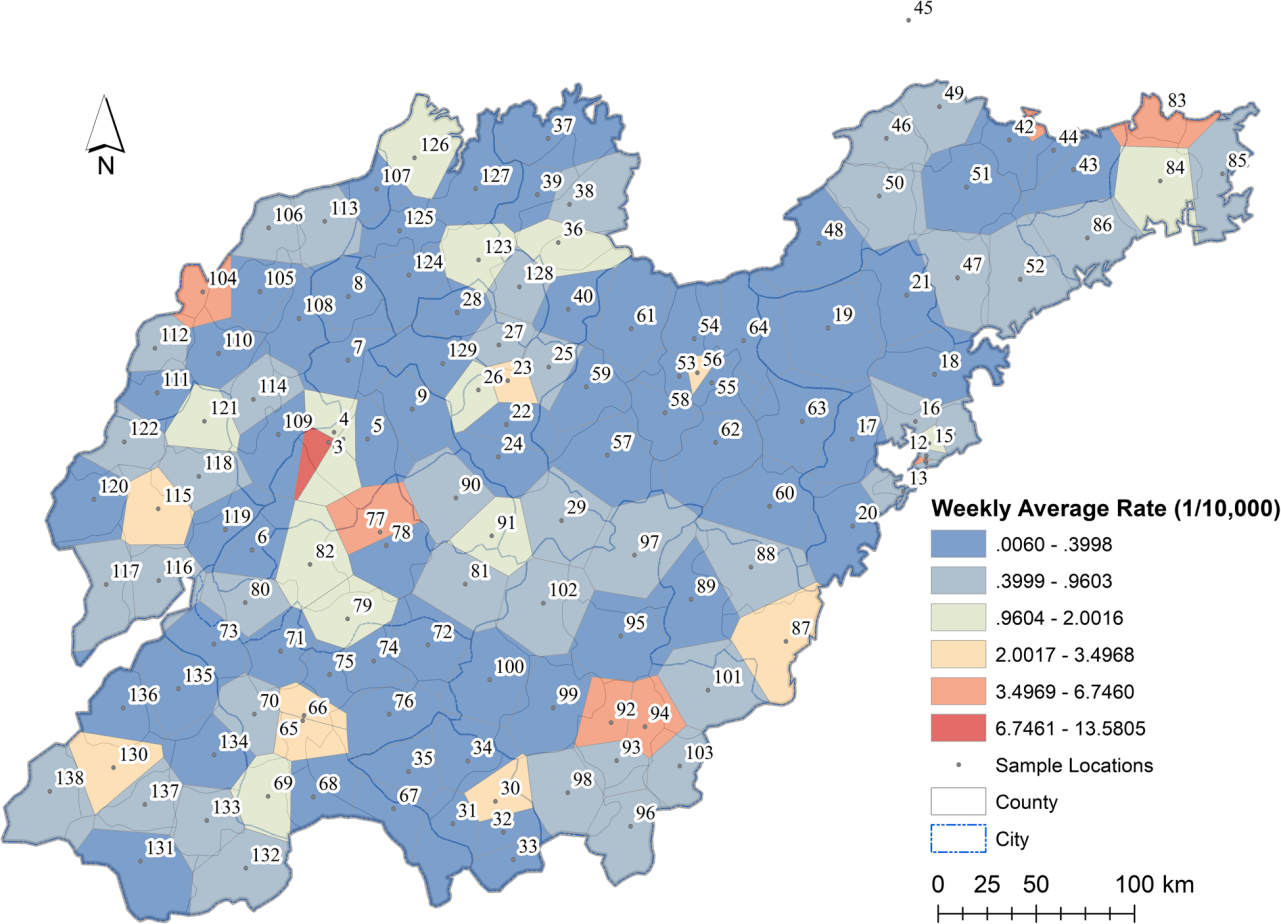
Location of Shandong province and weekly average incidence rates of HFMD in Thiessen polygons with 138 central sample locations. Shandong geographic database were provided by National Geomatics Center of China (http://www.ngcc.cn/ngcc/) at a 1:1,000,000 scale as the layer’s attribute. Thematic mapping was implemented in the ArcGIS platform (ESRI Inc)

Shandong is an eastern coastal province of China and is located between 34° 23′ and 38° 24′ north latitude and between 114° 48′ and 122° 42′ east longitude (Fig. 1). It extends to the Yellow Sea in the east and is bordered by the Hebei, Henan, Anhui and Jiangsu provinces from northwest to southwest. The Shandong province has a total population of approximately 100.47 million and a total land area of 157,100 km^2^. The gross domestic product (GDP) of Shandong province was 7646.97 billion Yuan in 2018. Shandong falls in the warm temperate monsoon climate zone, with an annual average temperature and precipitation in the ranges of 11–14 °C and 550–590 mm, respectively. More than 60% of the annual rainfall in the Shandong province is registered in the summer, and high temperatures usually occur in seasons with high precipitation.

### Data

From May 1st, 2008 to March 19th, 2009 (47 weeks), weekly HFMD incidence reports for a total of 138 districts in Shandong were collected from the Chinese Centre for Disease Control and Prevention. To reduce the influence of population size, weekly incidence rates were calculated to reflect the risk of the HFMD epidemic for sample locations, and the corresponding Thiessen polygons were constructed to account for spatial effects (Fig. 1). Monthly meteorological data from May 2008 to March 2009 were obtained from the China National Meteorological Information Center (http://data.cma.cn/), including the daily average, maximum, and minimum temperatures (°C), the air pressure (hPa), relative humidity (%), wind speed (m/s), precipitation (mm) and sunshine hours (h). The socioeconomic data were collected from the 2008 statistic Yearbook of Shandong province, including GDP (10,000 Yuan), ratio of the number of primary school students to the total population (%) and number of hospital beds per capita. *u*_1_–*u*_8_ and *u*_9_–*u*_11_ are used to denote the above eight meteorological factors and three socioeconomic factors, respectively. Spatial Kriging methods were used to calculate the weekly average meteorological factors for each sample location during the 47-week study of HFMD epidemics. Both dynamic meteorological factors and static socioeconomic factors were normalized to the range of 0–1.

### Geographically weighted regression model

Compared with the global multivariate regression model, local models can be more effective at describing potential local variations in relationships between dependent and independent variables. The geographically weighted regression [35, 36] is a typical local multivariate regression model extensively applied to measure the spatial relationships between variables and corresponding local variations across an entire area. Moreover, GWR model can clearly detect and interpret any non-stationary features of spatial patterns and associations, and has been widely used to estimate the epidemic risk and assess the influence of the epidemic determinants [37, 38]. The GWR model used in this study is as follows:

1 $$ {y}_i=\alpha \left({u}_i,{v}_i\right)+\sum \limits_k{\beta}_k\left({u}_i,{v}_i\right){x}_{k,i}+\sum \limits_l{\gamma}_l{z}_{l,i}+{\varepsilon}_i $$

where *y*_*i*_ is the HFMD incidence rate at location *i* with coordinates *u*_*i*_ and *v*_*i*_, *α* (*u*_*i*_, *v*_*i*_) is the corresponding intercept constant, *x*_*k,i*_ are a series of independent variables describing local variations, *β*_*k*_ (*u*_*i*_, *v*_*i*_) are the local regression coefficients to be estimated, which vary with location, *z*_*l,i*_ are a series of independent variables connected with the global stability, *γ*_*l*_ are the corresponding static coefficients, and ε_*i*_ indicates the estimation error.

To approximate the HFMD incidence rate of each sample location in Shandong province, we take the dynamic meteorological factors as the local variables *x*_*k*_ in the above GWR model, and the static socioeconomic factors as the global variables *z*_*l*_. Therefore, every location in the study area has a set of specific coefficients to reflect the associations between the HFMD incidence rate and the global or local variables. To solve the proposed GWR model, we apply a Gaussian distance-decay function to represent the relative importance between locations and an adaptive kernel scheme to determine the bandwidth (optimal number of neighboring locations), which is calculated through an iterative optimization process according to the Akaike Information Criterion (AIC). Meanwhile, the significance of the estimated global/local coefficients was checked with pseudo t tests and the model significance was tested by variance analysis (F tests).

### Kalman filter

The Kalman filter (KF) is a data fusion algorithm initially designed to solve the discrete-data linear filtering problem and provides a recursive solution to estimate the state variable of a time-varying system [39, 40]. In this study, KF is used to estimate the HFMD incidence and quantitatively assess the influence of risk factors. For a specific district, we define a multivariate state space *X*, which includes the HFMD incidence and several static socioeconomic factors. The state space is time-varying and calculated using the following parametric formula:

2 $$ {X}_t={AX}_{t-1}+{BU}_t+{\omega}_t $$

where *X*_*t*_ is the state vector containing the HFMD incidence and socioeconomic factors at time *t*, *A* is the state transition matrix indicating the effects of each state variable at time *t*-1 on the state vector at time *t*, *U*_*t*_ is a vector containing control variables which are dynamic meteorological and static socioeconomic factors relevant to this study, *B* is the control coefficient matrix indicating the effects of each control variable on the state vector, and *ω*_*t*_ is a random variable representing the process noise, which is drawn from a zero-mean Gaussian distribution *N*(0, *Q*). Last, *Q* stands for the prediction noise variance and accounts for the prediction uncertainty compared with the real process. The prediction of the time-varying state vector could be implemented as follows:

3 $$ {\hat{X}}_t={AX}_{t-1}+{BU}_t $$

where ${\hat{X}}_t$ is the prediction state vector at time *t* and *X*_*t*-1_ is the estimated (filtered) state vector at time *t*-1. The *a priori* estimation error covariance of the above prediction model propagates according to the equation:

4 $$ {\hat{P}}_t={AP}_{t-1}{A}^T+Q $$

where ${\hat{P}}_t$ is the estimation error covariance of the prediction model at time *t*. Furthermore, by considering the HFMD incidence *Y* as the most important variable in the state vector *X*, we define a simple linear relationship linking the measurement *Y* to the state vector *X*:

5 $$ {Y}_t={CX}_t+{v}_t $$

where *Y*_*t*_ is the measurement HFMD incidence at time *t* which is the observed incidence calculated based on the reported cases, *C* is the observation operator matrix, and *v*_*t*_ is a random variable representing the measurement noise which is also assumed to be drawn from a zero-mean Gaussian distribution *N*(0, *R*). Similarly, *R* stands for the measurement noise variance and represents the measurement uncertainty.

When both the process prediction and the measurement are considered, the *a priori* estimation error covariance ${\hat{P}}_t$ and the measurement noise variance *R* are combined to generate the Kalman gain:

6 $$ {K}_t={\hat{P}}_t{C}^T/\left(C{\hat{P}}_t{C}^T+R\right) $$

where *K*_*t*_ stands for the Kalman gain at time *t* and is applied to compute the *a posteriori* estimation of the state vector at time *t* as the following linear combination of the *a priori* estimation ${\hat{X}}_t$ and the actual measurement *Y*_*t*_:

7 $$ {X}_t={\hat{X}}_t+{K}_t\left({Y}_t-C{\hat{X}}_t\right) $$

As a function of the state vector covariance and the measurement noise, the Kalman gain *K*_*t*_ is noticeably high if the estimation error covariance is much higher than the measurement noise and the *a posteriori* estimation of the state vector significantly follows the measurements. Conversely, when *K*_*t*_ is low, the filter will essentially follow the predictions. In fact, *K*_*t*_ establishes the best combination between the process prediction and the measurement in order to minimize the mean square error between the *a posteriori* estimation *X*_*t*_ and its true value. After the update of the state vector as described above, the *a posteriori* estimation error covariance can be expressed as:

8 $$ {P}_t=\left(I-{CK}_t\right){\hat{P}}_t $$

where *I* is an identity matrix and *P*_*t*_ indicates the estimation error covariance after the prediction and the update at time *t*. The *a priori* estimations take place at each step of the recursive solution based on the last *a posteriori* estimations, according to Eqs. (3) and (4), the Kalman gain at each step is computed according to Eq. (6), and the *a posteriori* estimations which are also the *a priori* estimations of the next step are generated according to Eqs. (7) and (8). Beginning from the initial state, the prediction and the update appear at every single step of the KF recursive solution.

### Integration of the Kalman filter with the GWR model

Weekly averages of HFMD incidences in the sample locations were collected; the corresponding spatial autocorrelation was weak, with a Moran’s *I* of 0.0208 (*p* = 0.5460, calculated in ArcGIS Pro 2.3, https://pro.arcgis.com). However, the spatial stratified heterogeneity of the HFMD incidence among counties was statistically significant, with a GeoDetector *q*-statistic of 0.2153 (*p* < 0.001) [41, 42]. Therefore, GWR model was applied to explore the global or local associations between the HFMD incidence and meteorological or socioeconomic factors. Eight meteorological factors (*u*_1_–*u*_8_) were applied to be local (varying) variables and three socioeconomic factors (*u*_9_–*u*_11_) were used as global (fixed) variables. The GWR model to estimate the spatial distribution of HFMD incidences can be described as:

9 $$ {y}_i={\alpha}_i+\sum \limits_k{\beta}_{k,i}{x}_{k,i}+\sum \limits_l{\gamma}_l{z}_{l,i}+{\varepsilon}_i $$

where *y*_*i*_ is the incidence at location *i*, *α*_*i*_ is the intercept, and ε_*i*_ indicates the estimation error. *x*_*k,i*_ denote the local factors (*u*_1_–*u*_8_) and *β*_*k,i*_ are the varying coefficients of local meteorological variables at location *i*. *z*_*l,i*_ indicate the global factors (*u*_9_–*u*_11_) and *γ*_*l*_ are the static coefficients of global socioeconomic variables. For 138 monitored districts of the studied area, coefficients *γ*_*l*_ are constant, and coefficients *β*_*k,i*_ are organized into a matrix composed by 138 rows and 8 columns respectively.

**Fig. 2:**
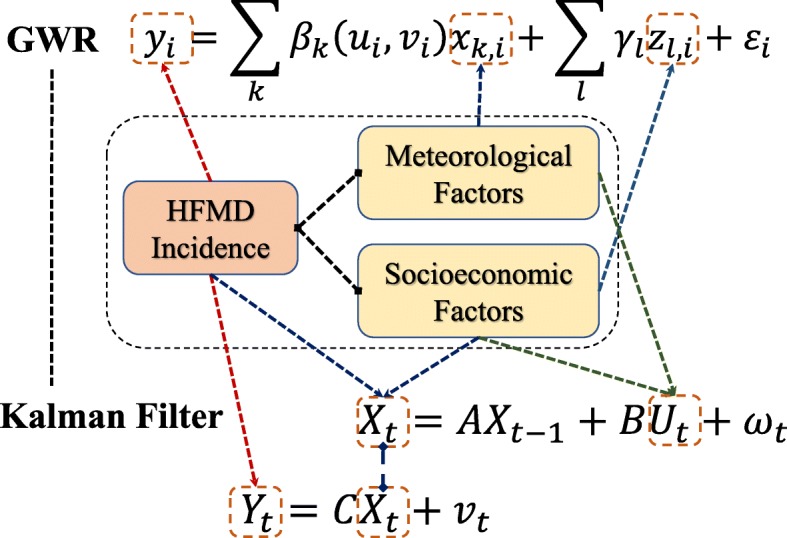
Integration framework of the Kalman Filter with the GWR model

The GWR analysis was accomplished in GWR 4.0.90 software (https://gwrtools.github.io/) and produced an overall coefficient of determination *R*^2^ of 0.2482, which was only an approximately 14% improvement compared with the global regression prediction. No significant local coefficients were found with an alpha level of 0.05 (see more in Additional file 1). Whereas specific corrections can optimize the coefficient significance of pseudo t test (e.g., a correction to avoid false positives in GWR [43]), there might be false negatives in our results. These were possibly caused by the measurement noise in the HFMD incidence, as well as the prediction noise of the GWR model. To better explore the spatiotemporal patterns and assess the determinant factors of the HFMD epidemic, we combined the Kalman filter with the GWR model (Fig. 2). The filtering allows to couple the measured and predicted incidences, and improve the incidence estimation accuracy. On the other hand, GWR model indicates the associations between incidence and determinant factors, and therefore could provide the prediction modeling of state vector varying in the Kalman filter. Notice that, during the time-varying process of Kalman filtering, the measurement and prediction would be combined together to improve the estimation accuracy recursively, and thus, the prediction effectiveness of the state space model derived by GWR is not pre-required. Furthermore, the influence sensitivity of the control variables can be evaluated during the incidence filtering process, and the corresponding determinants of HFMD incidence can be quantitively assessed.

In our proposed Kalman filter, the multivariate state vector *X* is composed by HFMD incidence (*y*) and static socioeconomic factors (*u*_9_–*u*_11_). *Y* indicates the measured incidence and the parameter *C* is a simple observation operator matrix that indicates the transition between the state vector *X* and the measured incidence *Y*. Notice that, the measurement *Y* can be a vector consist of multiple explained variables (e.g. incidence and prevalence), and therefore, Kalman filter is effective to solve multivariate multiple regression problems.

The state transition matrix *A* models the variation of the state vector that consists of the HFMD incidence and the static socioeconomic factors from time *t*-1 to time *t*. Coefficients *γ*_*l*_ derived from Eq. (9) indicate the global relationships between the incidence (*y*) and static factors (*u*_9_–*u*_11_). With a short 1-week timestep, the socioeconomic variables are constant along with the recursive solution. Thus, matrix *A* can be easily organized by an identity matrix and a row vector of coefficients *γ*_*l*_.

Both dynamic meteorological and static socioeconomic factors were selected as the vector containing controls in the Kalman filter. That is to say, vector *U* in Eq. (3) was consisted of meteorological factors (*u*_1_–*u*_8_) and socioeconomic factors (*u*_9_–*u*_11_). The control coefficient matrix *B* in Eq. (3) indicates the effects of each control variable on the state vector, and therefore, coefficients *β*_*k*,*i*_ derived from Eq. (9) could be applied to generate the parameter *B* in Eq. (3).

For different districts in the study area, the global and local effects of the determinant factors on the HFMD incidence vary spatially. Therefore, as shown in Fig. 2, we integrated the GWR model into the Kalman filter, derived the space-varying parameters *A* and *B*, and generated multiple filters for the various districts (138 filters for 138 districts). The integration has two implications: one is the variable correspondence and the other is the parameter transitivity. The HFMD incidence was the explained variable in GWR model, as well as the measurement *Y* in the Kalman filter. The local and global explanatory variables in the GWR model were the meteorological and socioeconomic factors, which also constitutes the control vector *U* of the Kalman filter. Moreover, the state vector *X* in the Kalman filter contains the HFMD incidence and the socioeconomic factors. For each district, the global coefficients *γ*_*l*_ and the local coefficients *β*_*k*,*i*_, which indicate the associations between the incidence and determinant factors, were obtained from the GWR result. Thus, the corresponding parameter *A* in the Kalman filter could be constructed from the global regression coefficients in the GWR model, while the parameter *B* using the local regression coefficients. Different from the parameters *A* and *C*, the control coefficient matrix *B* is district-dependent (various *B*s for districts), and the corresponding multiple filters describe the spatial variation of the HFMD incidence evolution patterns and determinant influence effects.

During the recursive filtering process, the prediction ${\hat{X}}_t$ at time *t* was calculated by the state space model with the estimation *X*_*t*-1_ at time *t*-1 according to Eq. (3), and the *a priori* estimation error covariance at time *t* was calculated with the *a posteriori* one at time *t*-1 according to Eq. (4). The Kalman gain *K*_*t*_ at time *t* could be derived from the *a priori* estimation error covariance and the measurement noise variance according to Eq. (6). And the *a posteriori* estimation *X*_*t*_ at time *t* was updated according to Eq. (7), which indicates the estimated HFMD incidence of a specific district at time *t*. The corresponding *a posteriori* estimation error at time *t* could be calculated according to Eq. (8). After the time-varying recursion, the estimations and errors of HFMD incidences of all sample locations could be implemented.

## Results

### Kalman filtering validation

The HFMD incidence rates of 138 monitored districts were obtained in 47 weeks (from May 1st, 2008 to March 19th, 2009). For each of the sample districts continuous weekly incidence rates were available, the week index varying from 1 to 47. The average weekly incidence by district varied with time and had a mean value of approximately 0.936 × 10^− 4^ (in a range of 0.043 × 10^− 4^–4.851 × 10^− 4^). Eight meteorological factors (air pressure, daily average, maximum, and minimum temperatures, precipitation, relative humidity, wind speed and sunshine hours) were selected as the local dynamic independent variables (*u*_1_–*u*_8_), and the global static independent variables (*u*_9_–*u*_11_) were the following three socioeconomic factors: GDP, ratio of primary school students and number of hospital beds per capita. Both dynamic and static variables were normalized to the range of 0–1.

**Fig. 3:**
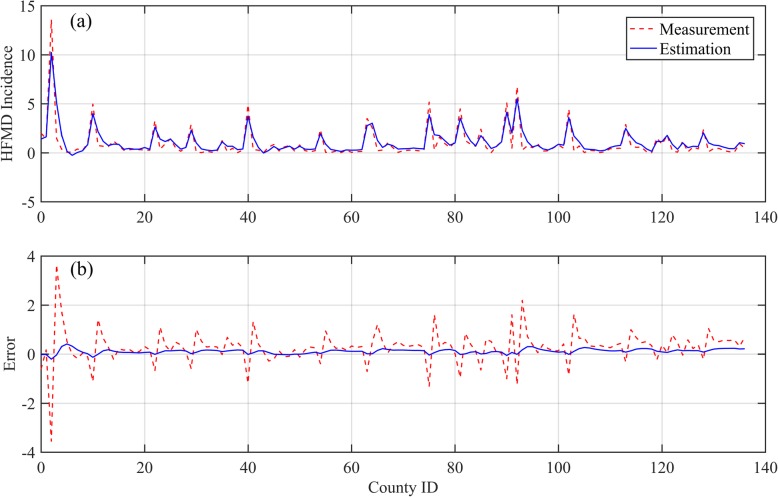
Efficiency evaluation of the Kalman Filter for the HFMD incidence assessment. (**a**) Measurement and estimation incidences. (**b**) Measurement and estimation errors

**Fig. 4:**
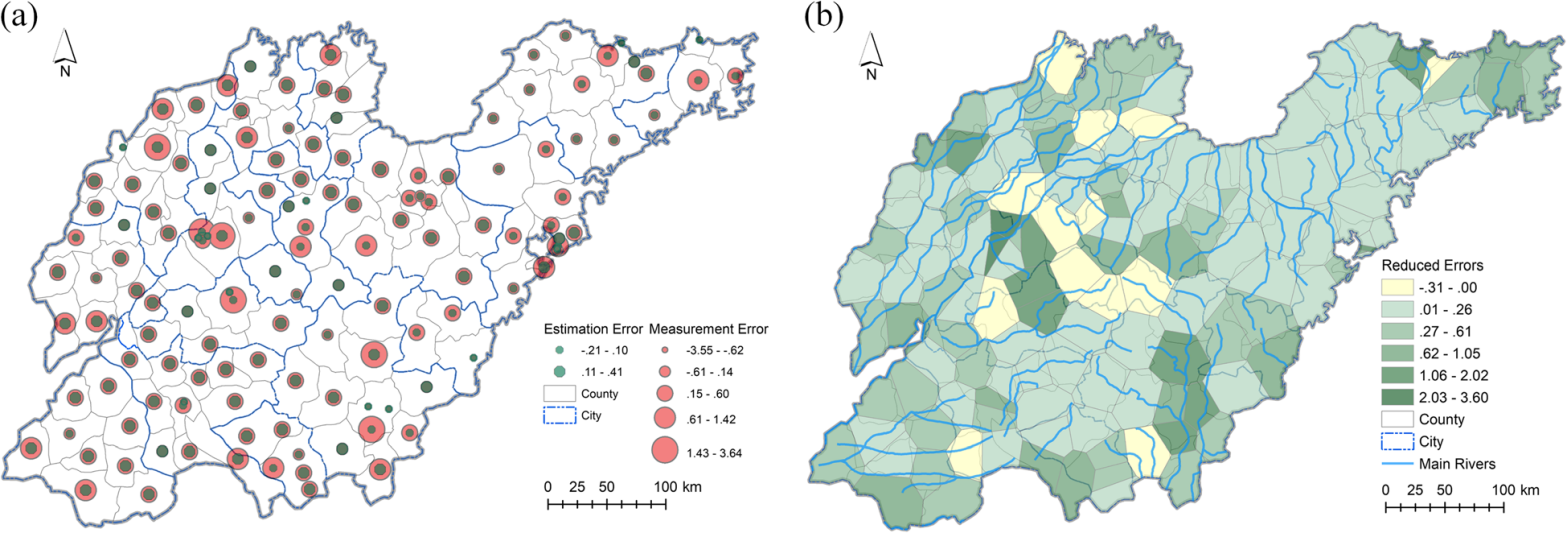
Error distributions of the global HFMD incidence filtering. (**a**) Measurement and estimation errors. (**b**) Reduced errors. Shandong geographic database were provided by National Geomatics Center of China (http://www.ngcc.cn/ngcc/) at a 1:1,000,000 scale as the layer’s attribute. Thematic mapping was implemented in the ArcGIS platform (ESRI Inc)

To evaluate the overall efficiency of the Kalman filter for HFMD incidence assessment, weekly incidence rates and meteorological variables for the studied districts were first aggregated to weekly average values. Next, using the static socioeconomic variables, the regression coefficients were calculated with the ordinary least squares (OLS) linear regression method. Subsequently, these coefficients were applied to generate the parameters *B* and *C* within the Kalman filter model, and the initial prediction and measurement errors were assumed to be drawn from a standardized Gaussian distribution. As shown in Fig. 3a, the filtering provided an adjustment to the weekly average HFMD incidences in the 138 districts to some extent compared to the corresponding measured values, and the estimated HFMD incidences followed a similar distribution as the measurements. Figure 3b illustrates that the original measurement errors varied among districts, high-value errors correlating to districts with high-value measurements; the estimation errors after filtering only apparently approach zero (the blue error curve presents an approximately horizontal line around the x axis). Even in districts with high-value measured incidence, the Kalman filter satisfactorily reduces the estimation errors. The measurement and estimation errors of the HFMD incidences in districts are mapped in Fig. 4a. The HFMD incidence errors were reduced from the range of − 3.55 × 10^− 4^–3.64 × 10^− 4^ to − 0.21 × 10^− 4^–0.41 × 10^− 4^. The Kalman filter significantly reduced the incidence errors for the majority of the districts, especially for those with large measurement errors. Figure 4b illustrates the reduced error distribution after filtering: although several districts received negative error reductions, the errors that increased were small and approximately 10% of the reduced ones. Regions with large error reductions and large HFMD incidences had similar reduced error distributions and were surrounded by regions with negative error reductions (the light-yellow polygons surround the dark-green ones). Overall, the Kalman filter plays an effective role for HFMD incidence assessment even if the filter parameters are derived from the OLS linear regression without spatial variances. The measurement error covariance was 0.5686, whereas the estimation error covariance was substantially reduced to 0.0211 after filtering.

### The spatiotemporal pattern of HFMD incidence filtering

**Fig. 5:**
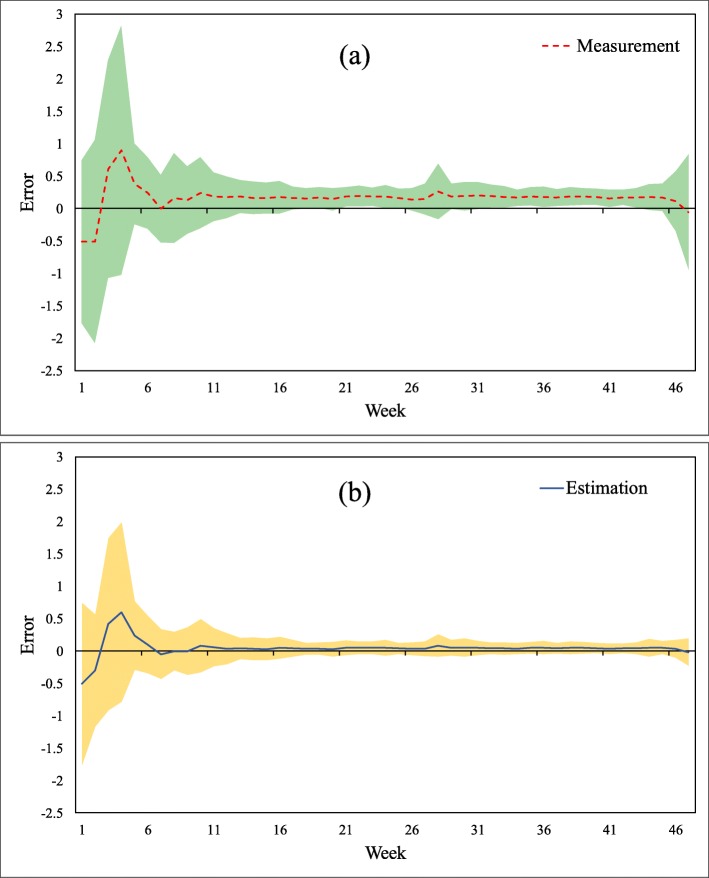
Temporal variation of the average errors of filtered HFMD incidence by district (the shaded area denotes the interval of 1 standard deviation around the average error). (**a**) Measurement errors. (**b**) Estimation errors

After the overall validation of the Kalman filtering for the HFMD incidence assessment, we applied this model to explore the spatiotemporal patterns of HFMD incidences for all 138 districts. The local and global coefficients of dynamic meteorological factors (*u*_1_–*u*_8_) and static socioeconomic factors (*u*_9_–*u*_11_) on the HFMD incidences of each district were separately calculated using the GWR model. The corresponding parameter *B* of the Kalman filter is a matrix array that includes 138 control coefficient matrices ([11 × 4]), indicating the effects of meteorological and socioeconomic factors (*u*_1_–*u*_11_) on the state vector for 138 districts, respectively. A total of 138 Kalman filters with spatial variations were used to assess the temporal changes of HFMD incidences in the studied districts under the determinant factors (*u*_1_–*u*_11_). As shown in Fig. 5a, the average errors of measured incidences started with a high initial value and varied from week 1 to week 47; the error interval of 1 standard deviation (1-StdDev) around the average showed local fluctuations, which are probably related to the abnormal temporal intervals of the HFMD incidence evolution. For instance, there was a tiny error increase that appeared in the 28th week (beginning on November 6th) accompanying a substantial interval expansion; the error intervals expanded significantly even when the error mean decreased to nearly zero in weeks 46 and 47 (beginning on March 12th). Figure 5b shows that, compared to the measurements, the error means and 1-StdDev intervals of measurement incidences were reduced. However, considering the above-mentioned temporal anomalies, even after filtering the error means and 1-StdDev intervals were still large in the first 8 weeks (beginning on May 1st). That is to say, the HFMD epidemic in Shandong probably had pronounced seasonality features, usually evolving from mid-March, increasing until late June and with a potential reversal in early November.

**Fig. 6:**
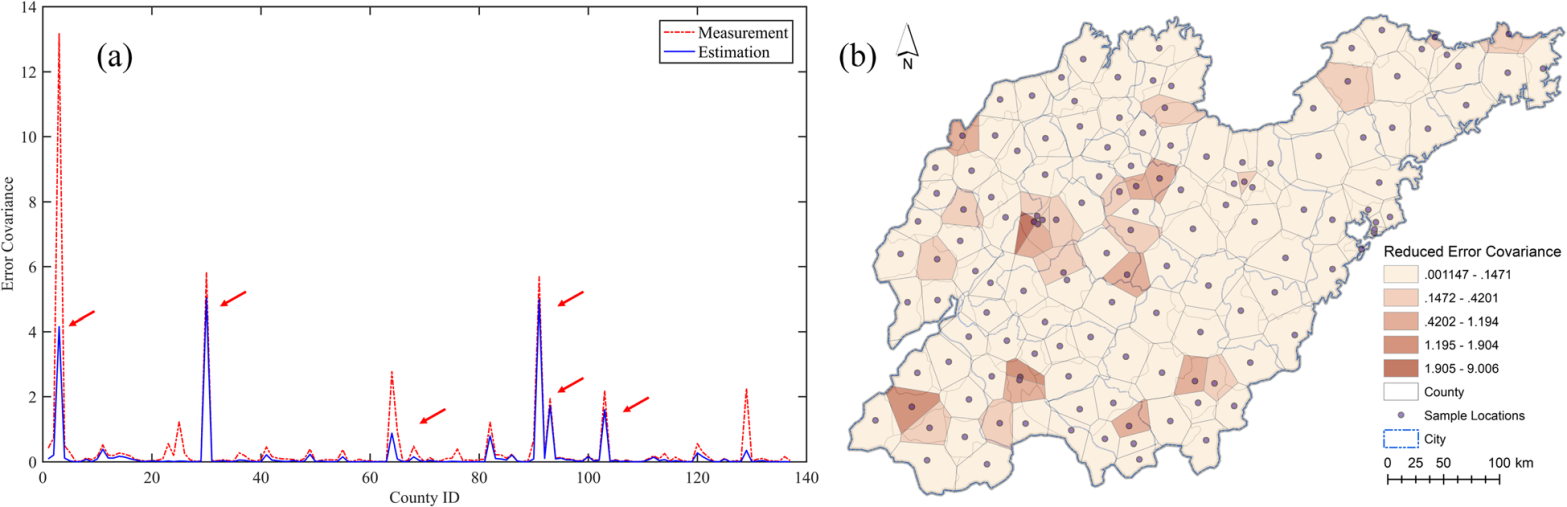
Spatial variation of error covariances of HFMD incidence filtering in districts. (**a**) Measurement and estimation covariances. (**b**) Reduced covariance distribution. Shandong geographic database were provided by National Geomatics Center of China (http://www.ngcc.cn/ngcc/) at a 1:1,000,000 scale as the layer’s attribute. Thematic mapping was implemented in the ArcGIS platform (ESRI Inc)

To explore the spatial variation of the HFMD incidence filtering, the error covariances of incidence measurements and estimations by district were analyzed as shown in Fig. 6a. The majority of districts had satisfactory reductions of error covariances after filtering and several districts received noticeable reductions even when the original error covariances were large. However, the error covariances of several districts were still significant after filtering, and the Kalman filters played a weak role in these districts (their positions are indicated by red arrows). Figure 6b illustrates the spatial variation of the reduced error covariances by district after HFMD incidence filtering. The average error covariance of measured incidences was 0.3841, whereas the average estimated incidence error covariance was reduced to 0.1846, indicating an overall improvement of over 50% error reduction. However, several districts with significant error reductions overlapped to a certain extent with districts of large estimated incidence error covariances (Fig. 6). In other words, the HFMD incidence evolutions in these districts were abnormal, deeming such areas as potential risk regions of HFMD epidemic outbreaks.

**Fig. 7:**
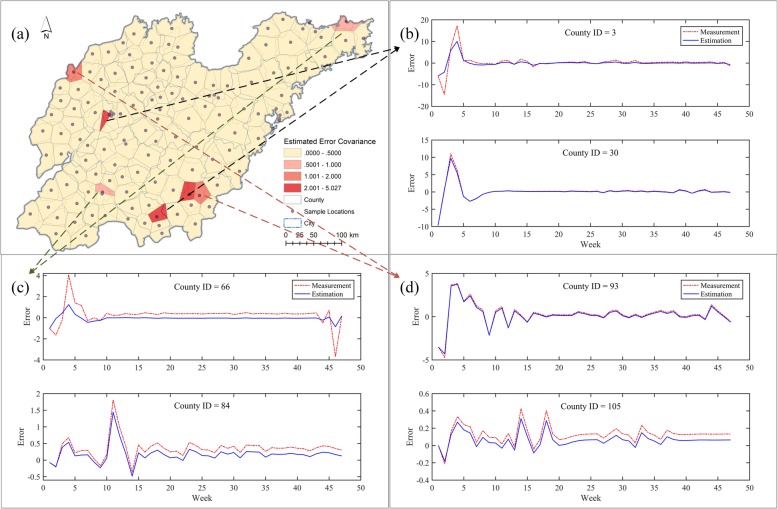
Potential HFMD risk regions and the separate error variations of incidence filtering. (**a**) Distribution of risk regions. Error variations of incidence filtering of (**b**) 1st category; (**c**) 2nd category; (**d**) 3rd category. Shandong geographic database were provided by National Geomatics Center of China (http://www.ngcc.cn/ngcc/) at a 1:1,000,000 scale as the layer’s attribute. Thematic mapping was implemented in the ArcGIS platform (ESRI Inc)

Further considerations were proposed in these specific districts, and among them (Fig. 6b), error covariances of estimated incidences were classified in natural breaks and mapped in Fig. 7a. Judging by the temporal variations of the filtered HFMD incidence errors in each district, three classes of potential risk areas were distinguished, and presented separately in Fig. 7b, c and d, respectively. The temporal measurement curves and the estimation errors in two districts of the same class were extremely similar to each other. Although the spatial aggregation feature of these abnormal districts was weak (Fig. 7a), we could still classify potential HFMD risk regions into three categories by using the Kalman filter model in association with the meteorological and socioeconomic factors. As shown in Fig. 7b, the error curves of HFMD incidence filtering greatly varied in the early period but maintained a long-term steady trend. The second type of potential risk regions is illustrated in Fig. 7c; such regions present a relatively long-term steady trend with slight variations within a few intervals. Last, the third type had significant oscillations during the long-term period and unsteady oscillations appear in unpredictable localized time intervals (Fig. 7d). Evidently, the former two types of potential HFMD risk regions raise concerns during the localized periods, especially in HFMD high-incidence seasons. Although the risk regions of the latter type were probably characterized by relatively low incidences, the HFMD epidemic evolutions were unsteady in the long-term, thus more prevention and control policies (e.g. long-term epidemic surveillance) should be implemented in these specific districts. Overall, the proposed HFMD incidence filtering in Shandong showed a strong seasonal dependence and several specific potential HFMD risk regions were found without significant spatial clustering.

### Influence sensitivity of determinant factors

The control coefficient matrix *B* of the Kalman filter was generated from the GWR results to indicate the relationships between the HFMD incidence and meteorological or socioeconomic factors. To assess the influence that each factor has on the HFMD incidence, we defined an index *ζ*_*j*_ (*j* = 1–11) to describe the assumed enhancement effect of determinant factors (*u*_1_–*u*_11_). Experiments were repeated to evaluate the influence sensitivity of each dynamic or static factor on the HFMD incidence filtering. In experiment *j*, *ζ*_*j*_ varied from 0 to 5 with a step of 0.5, which indicates that the enhancement effect of factor *u*_*j*_ had a step size of 50% increase, while the *ζ*_*i*_ (*i* ≠ *j*) of other factors was kept invariant. The average errors and covariances of incidence estimations by district were applied to assess the influence sensitivity of meteorological and socioeconomic factors.

**Fig. 8:**
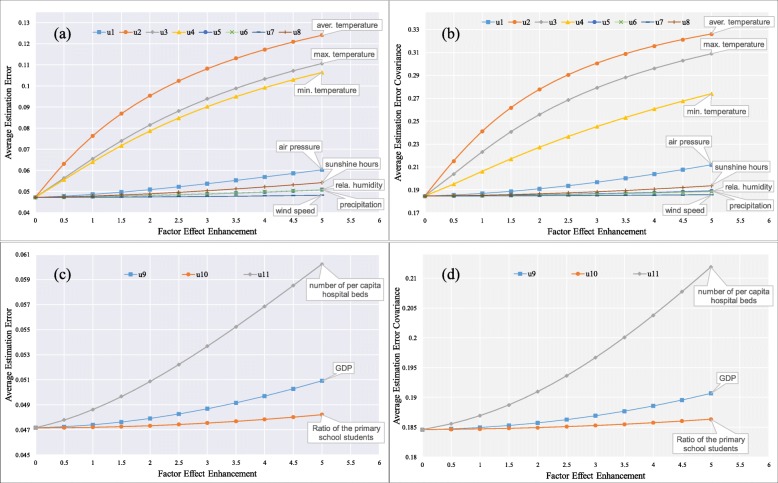
Influence sensitivity of HFMD incidence filtering along with meteorological and socioeconomic factors. (**a**) Average errors and (**b**) covariances of meteorological factors (*u*_1_–*u*_8_). (**c**) Average errors and (**d**) covariances of socioeconomic factors (*u*_9_–*u*_11_)

Figure 8a and b demonstrate the variations of the average estimation errors and covariances of HFMD incidence filtering along with the variation of each meteorological factor. As expected, the temperature factors (*u*_2_–*u*_4_) played the most important roles in the relationship with HFMD incidence filtering, and the average estimation errors and covariances were both sensitive to their enhancement effects, suggesting that higher temperature variations would cause a higher HFMD variation. Air pressure (*u*_1_) was a secondary determinant affecting the HFMD variation approximately 25% as strongly as the temperature factors (Table 1). The next secondary determinants were sunshine hours, relative humidity, and precipitation (*u*_8_, *u*_6_, *u*_5_). Compared to the latter rainfall factors, the effect of sunshine hours on the HFMD incidence variation was almost twice as much (Table 1). As shown in Fig. 8a and b, the wind speed (*u*_7_) played a very weak role in HFMD incidence filtering, with a relative variation of nearly zero, reflecting that the HFMD epidemic probably had little airborne contagious transmission. Figure 8c and d illustrate the influence of socioeconomic factors on the HFMD incidence filtering. The number of hospital beds per capita (*u*_11_) was the dominant determinant, followed by the GDP (*u*_9_), which influenced the HFMD incidence approximately 30% as strongly as the dominant factor (Table 1). The relative variation of HFMD incidence filtering with the ratio of primary school students (*u*_10_) was very slight (Table 1), suggesting that the amount of susceptible population in the studied region was probably not the leading cause of the HFMD variation. Overall, the daily average, maximum, and minimum temperatures and air pressure were the dominant meteorological factors, while the number of hospital beds per capita and GDP were the dominant socioeconomic ones that influenced the HFMD incidence variation in Shandong. Concomitantly, the HFMD variation was extremely slight even at high values of the wind speed and ratio of primary school students.

**Table 1:** Relative variations of average errors and covariances of HFMD incidence filtering with meteorological and socioeconomic factors

Determinant factors	Variable	Relative variation (%)	
		Average error	Average covariance
Air pressure (hPa)	*u*_1_	4.95	2.78
Daily average temperature (°C)	*u*_2_	21.05	11.92
Daily maximum temperature (°C)	*u*_3_	18.05	10.65
Daily minimum temperature (°C)	*u*_4_	17.14	8.07
Precipitation (mm)	*u*_5_	1.48	0.48
Relative humidity (%)	*u*_6_	1.49	0.42
Wind speed (m/s)	*u*_7_	0.44	0.12
Sunshine hours (h)	*u*_8_	2.77	0.96
Gross domestic product (GDP) (10^4^ CNY)	*u*_9_	0.65	1.54
Ratio of primary school students (%)	*u*_10_	0.19	0.44
Number of hospital beds per capita	*u*_11_	2.78	4.95

## Discussion

In recent years, Kalman filters have been extensively used in a variety of applications, such as land cover classification [44] or landslide susceptibility evaluation [45]. Typical applications in Earth science concentrate on remote sensing image processing [44, 46–49] and data assimilations in the fields of agriculture [50–52], agrology [53, 54], ecology [55], hydrology [56, 57], oceanography [58] and others. In epidemiology, Kalman filters are usually applied to the mathematical modeling of epidemic spreads for diseases such as HIV/AIDS and Ebola [59–61]. In the present study, a Kalman filter was used to estimate the spatiotemporal evolution of HFMD incidence in 138 districts of the Shandong province, China, by integration with a GWR model to identify the local relationships between the HFMD incidence and risk factors. The proposed integrated model showed significant improvement in the HFMD incidence estimation accuracy. The spatiotemporal variation characteristics and potential risk regions of HFMD incidence were explored, and the influence of meteorological and socioeconomic factors on the HFMD variation were assessed. The results showed that the Kalman filter was effective for the HFMD incidence assessment in Shandong and produced a reduction of error covariance from 0.5686 to 0.0211 at the provincial scale. Considering the spatial variation of Kalman filters for various districts, the error covariance was reduced from 0.3841 to 0.1846 after filtering. Furthermore, filter processing allowed to identify potential HFMD risk regions: three categories of risk regions could be distinguished, with manifest filtering oscillations in the initial, local and long-term periods, respectively. Although the detected potential risk regions did not exhibit significant spatial clustering, more attention should be paid to these districts, especially the ones in the third category, with long-term filtering oscillations.

In addition to exploring the HFMD spatiotemporal patterns, the influence sensitivity of meteorological and socioeconomic factors was determined. We found that three temperature factors were the dominant meteorological determinants of the HFMD epidemic in Shandong, although the air pressure also affected the HFMD epidemic to a certain extent; however, wind speed had no manifest effect. Intense variations of temperature or air pressure produced high variations of HFMD incidence, whereas the influence of wind speed on the epidemic incidence was negligible and unclear. The HFMD related viruses are probably sensible to temperatures and air pressure. The main transmission routes of HFMD epidemic are the intimate contacts, and the wind speed influences slightly to the epidemic spread. With an overall shortage of rainfall in Shandong province, the HFMD epidemic spread might have less sensitivity to precipitation and relative humidity in a low-value level. Our findings are consistent with a number of previous studies [13, 18, 25, 26, 62, 63]. The environmental temperature relates to behavioral patterns such as increased contact among young children, thereby facilitating the spread of an HFMD infection [16]. However, our results indicate that meteorological factors such as precipitation, relative humidity and sunshine hours were not strongly associated with HFMD incidence, which is partially inconsistent with some of the previous studies. For instance, precipitation was strongly correlated with HFMD incidence in Singapore [25], and the number of HFMD cases increased significantly with increasing relative humidity in Japan [26]. HFMD cases at the county level across mainland China were spatially clustered and closely linked to the amounts of monthly precipitation in the region [24]. Relative humidity and precipitation were also found as the dominant driving factors of HFMD incidence in Henan, China [16]. Moreover, compared to GDP and ratio of primary school students to the total population, the number of hospital beds per capita appeared to be more dominant in HFMD incidence in Shandong. The children behavior patterns were possibly consistent amongst the districts with various economic levels around Shandong province. The influence of the population density background was already partially reduced during the calculation The HFMD incidence. The healthcare level played a manifest role in the controls of the HFMD epidemic spread in Shandong province. This result differs from other studies as well. For instance, GDP was the primary risk factor contributing to the spatial distribution of HFMD incidence in Sichuan and Henan, China [13, 16]. Possible reasons for this discrepancy include the differences between the studied regions, different transmission mechanisms of the HFMD epidemics, seasonal variations of meteorological factors, scale effects, zoning effects and others.

This study provides a multi-perspective on estimating the spread of an HFMD epidemic by combining measurement noise with prediction uncertainty and demonstrates a novel approach to exploring the spatiotemporal patterns and determinant factors of an HFMD epidemic. Nevertheless, there are several limitations to this study, described as follows. First, we generated the basic local associations between the HFMD incidence and meteorological and socioeconomic factors using a GWR model without considering an HFMD mathematical model. Also, a limited number of driving factors were selected, which could have led to an insufficient description and interpretation of the HFMD epidemic dynamic mechanism. Second, our method was trained on county-level data from the Shandong Province of China from 2008 to 2009, and applied only for the pattern exploration and risk assessment of the HFMD epidemic. This approach could easily be extended to other regions and infectious diseases similar to HFMD, although it should be accompanied by a thorough analysis and benchmarking of the model on the new problem. Lastly, it was hypothesized that the measurement and prediction noises of the Kalman filter followed a zero-mean Gaussian distribution, and the model of the state vector and control variables was linear. These assumptions might have limited the applicability of the model; appropriate improvements could include non-linear filters and non-Gaussian noise distributions such as an extended Kalman filter (EKF), an unscented Kalman filter (UKF), or a particle filter (PF).

## Conclusion

This study introduces a novel perspective to explore the spatiotemporal patterns and determinant factors of an HFMD epidemic. To this purpose, a Kalman filter method integrated with the GWR model with the aim to identify the global and local relationships between HFMD incidence and dynamic meteorological and static socioeconomic factors was designed. The proposed method considers both measurement noise and prediction uncertainty, which reduces the estimation error covariance of the HFMD incidence and improves the estimation accuracy. The filter processing could help explore the spatiotemporal patterns and determinants of the HFMD epidemic. As a result, three specific categories of potential risk regions of HFMD epidemics in Shandong were identified, with temperature factors and number of hospital beds per capita as the dominant determinants of the epidemic incidence. Furthermore, our approach can be extended to other regions and other infectious diseases similar to HFMD.

## Supplementary information

**Additional file 1.** Details of the GWR analysis.

## Data Availability

The meteorological data used in the study are publicly available from the China National Meteorological Information Center (http://data.cma.cn/). The Socioeconomic data used in the study are publicly available from the statistical Yearbook of Shandong province (http://tjj.shandong.gov.cn/). The Shandong HFMD dataset is owned by the Chinese Centre for Disease Control and Prevention (http://www.chinacdc.cn/) and not available for distribution due to the constraint in the consent. The other dataset are available from the corresponding author on reasonable request.

## Abbreviations

CDC: Chinese Center for Disease Control and Prevention
EKF: Extended Kalman filter
GDP: Gross domestic product
GWR: Geographically weighted regression
HFMD: Hand, foot and mouth disease
KF: Kalman filter
NDVI: Normalized difference vegetation index
OLS: Ordinary least squares
PF: Particle filter
StdDev: Standard deviation
UKF: Unscented Kalman filter
