# Supplementary material for: Integration of a Kalman filter in the geographically weighted regression for modeling the transmission of hand, foot and mouth disease
Source: BMC Public Health. 2020 Apr 10;20:479. doi: 10.1186/s12889-020-08607-7 (PMC7146977; doi:10.1186/s12889-020-08607-7)
Supplement: Supplementary file 1 — Additional file 1. Details of the GWR analysis. [file 12889_2020_8607_MOESM1_ESM.docx]

**Appendix 1.** Details of the GWR analysis

**Table S1**. GWR model settings.

| **Setting & Option** | **Content** |
| --- | --- |
| Dependent variable | *y*: HFMD weekly average incidence (1/10^4^) |
| Local (varying) variables | *u*_1_: Air pressure (hPa) |
|  | *u*_2_: Daily average temperature (°C) |
|  | *u*_3_: Daily maximum temperature (°C) |
|  | *u*_4_: Daily minimum temperature (°C) |
|  | *u*_5_: Precipitation (mm) |
|  | *u*_6_: Relative humidity (%) |
|  | *u*_7_: Wind speed (m/s) |
|  | *u*_8_: Sunshine hours (h) |
| Global (fixed) variables | *u*_9_: Gross domestic product (GDP) (10^4^ CNY) |
|  | *u*_10_: Ratio of primary school students (%) |
|  | *u*_11_: Number of hospital beds per capita |
| Model type | Gaussian |
| Geographic kernel | Fixed Gaussian (distance) |
| Bandwidth selection | Golden section search |
| Selection Criteria | AICc |
| Coefficient significance | pseudo t test |
| Model significance | variance analysis (F test) |

**Table S2**. Summary of the GWR and global OLS results.

| **Parameter** | **GWR** | **Global OLS** |
| --- | --- | --- |
| Best bandwidth size | 345.049 km | – |
| -2 log-likelihood | 486.2173 | 488.1199 |
| Classic AIC | 514.3131 | 514.1199 |
| AICc | 517.7799 | 517.0792 |
| BIC/MDL | 555.3328 | 552.0796 |
| CV | 2.951211 | 2.9252 |
| R square | 0.2482 | 0.2180 |
| Adjusted R square | 0.1474 | 0.1423 |
| †The ANOVA comparison result (F=1.1929) showed no significant improvement with an alpha level of 0.05. | | |

**Table S3**. Summary statistics of GWR global/local coefficients.

| **Local coef.** | **Minimum** | **25% quartile** | **50% quartile** | **75% quartile** | | **Maximum** |
| --- | --- | --- | --- | --- | --- | --- |
| Intercept | 0.7831 | 0.8379 | 0.8906 | 0.9082 | | 0.9442 |
| *u*_1_ | 0.0189 | 0.1706 | 0.2184 | 0.2510 | | 0.3002 |
| *u*_2_ | -2.2534 | -0.5414 | 0.7586 | 3.3534 | | 5.9315 |
| *u*_3_ | -3.9316 | -2.1405 | -0.3171 | 0.5565 | | 1.7692 |
| *u*_4_ | -3.9245 | -2.3582 | -0.9461 | -0.1540 | | 0.8072 |
| *u*_5_ | -0.1683 | -0.1059 | -0.0608 | 0.0730 | | 0.2453 |
| *u*_6_ | -0.2982 | -0.0590 | 0.0110 | 0.0716 | | 0.2351 |
| *u*_7_ | -0.0267 | 0.0309 | 0.0540 | 0.1354 | | 0.2535 |
| *u*_8_ | -0.3592 | -0.2676 | -0.1503 | -0.1185 | | -0.0839 |
| **Global coef.** | **Estimate** | | **Standard Error** | | **Significance** | |
| *u*_9_ | 0.1213 | | 0.2020 | | *p* > 0.05 | |
| *u*_10_ | 0.0674 | | 0.1822 | | *p* > 0.05 | |
| *u*_11_ | 0.6953** | | 0.2123 | | *p* < 0.01 | |
| †No significant local coefficients were found with an alpha level of 0.05. | | | | | | |


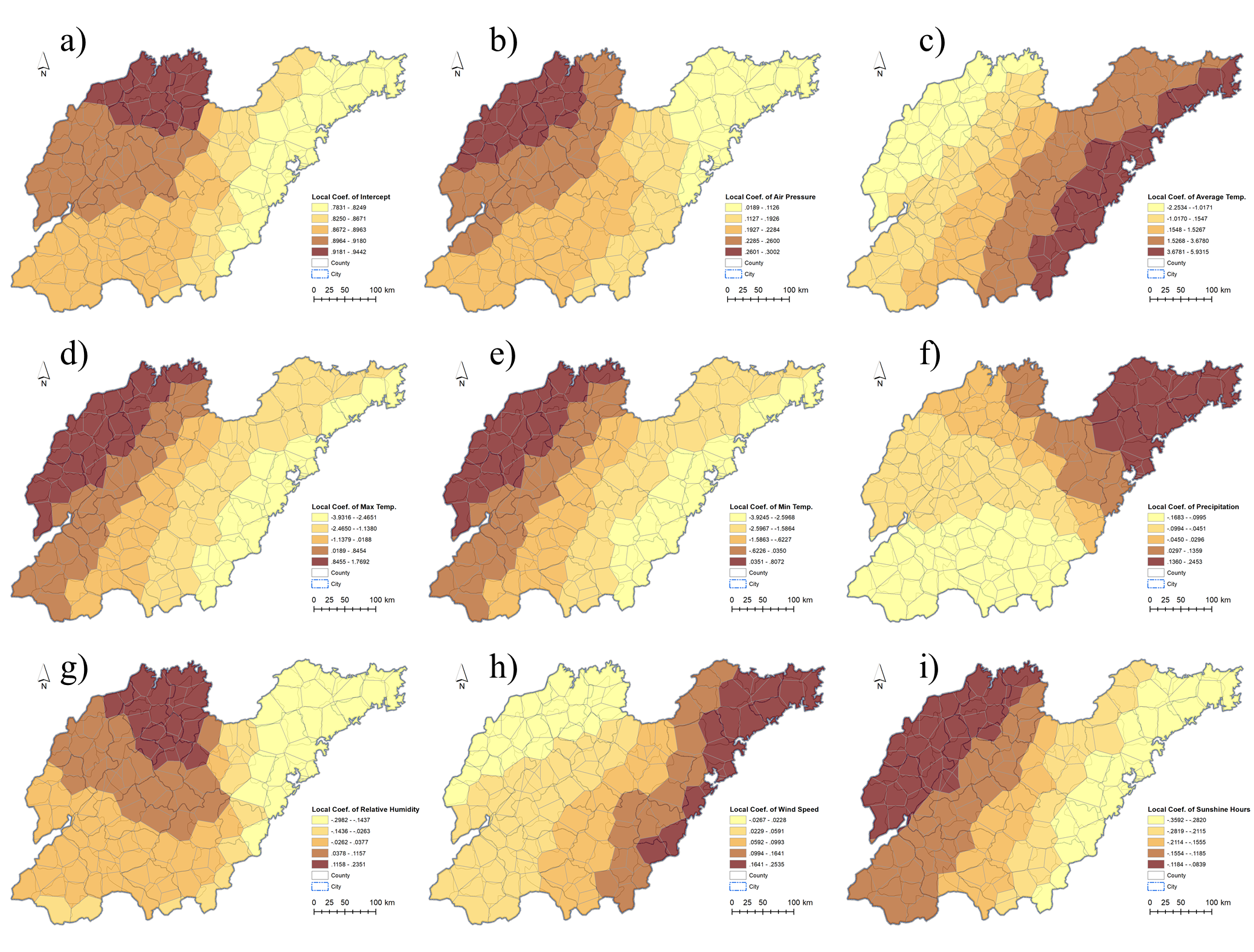


**Figure S1**. Spatial distributions of estimated parameters of GWR model. (a) Intercept; (b) *u*_1_; (c) *u*_2_; (d) *u*_3_; (e) *u*_4_; (f) *u*_5_; (g) *u*_6_; (h) *u*_7_; (i) *u*_8_. Shandong geographic database were provided by National Geomatics Center of China (<http://www.ngcc.cn/ngcc/>) at a 1:1,000,000 scale as the layer’s attribute. Thematic mapping was implemented in the ArcGIS platform (ESRI Inc).


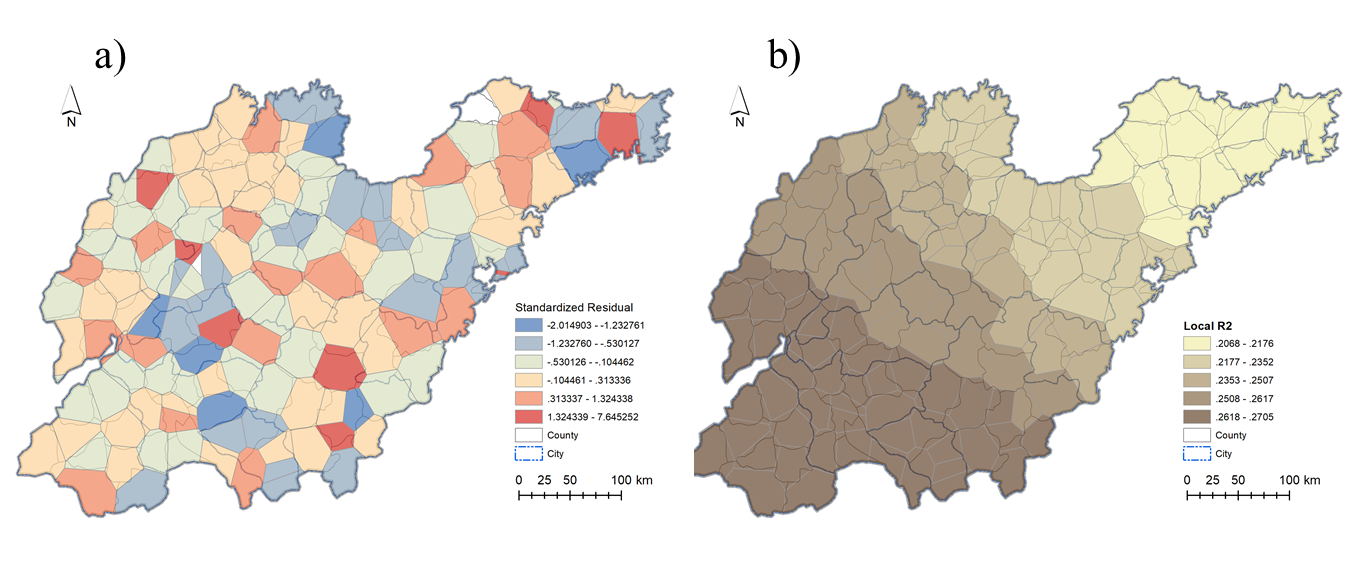


**Figure S2**. Spatial distributions of the GWR results. (a) Standardized residuals. (b) Local R square values. Shandong geographic database were provided by National Geomatics Center of China (<http://www.ngcc.cn/ngcc/>) at a 1:1,000,000 scale as the layer’s attribute. Thematic mapping was implemented in the ArcGIS platform (ESRI Inc).
